# Intestinal Microbiota and the Innate Immune System – A Crosstalk in Crohn’s Disease Pathogenesis

**DOI:** 10.3389/fimmu.2015.00489

**Published:** 2015-09-22

**Authors:** Lea-Maxie Haag, Britta Siegmund

**Affiliations:** ^1^Division of Gastroenterology, Infectious Diseases and Rheumatology, Medical Department 1, Charité – Universitätsmedizin Berlin, Berlin, Germany

**Keywords:** Crohn’s disease, innate immune system, inflammation, microbiota, dysbiosis

## Abstract

Crohn’s disease (CD) is a chronic, relapsing inflammatory disorder that can occur anywhere along the gastrointestinal tract. The precise etiology of CD is still unclear but it is widely accepted that a complex series of interactions between susceptibility genes, the immune system and environmental factors are implicated in the onset and perpetuation of the disease. Increasing evidence from experimental and clinical studies implies the intestinal microbiota in disease pathogenesis, thereby supporting the hypothesis that chronic intestinal inflammation arises from an abnormal immune response against the microorganisms of the intestinal flora in genetically susceptible individuals. Given that CD patients display changes in their gut microbiota composition, collectively termed “dysbiosis,” the question raises whether the altered microbiota composition is a cause of disease or rather a consequence of the inflammatory state of the intestinal environment. This review will focus on the crosstalk between the gut microbiota and the innate immune system during intestinal inflammation, thereby unraveling the role of the microbiota in CD pathogenesis.

## Introduction

Crohn’s disease (CD) is a chronic, relapsing inflammatory disorder affecting the gastrointestinal tract and together with ulcerative colitis (UC) commonly included in the collective term inflammatory bowel diseases (IBD). Inflammation associated with CD is characterized by a discontinuous, transmural pattern, and can affect any part of the gastrointestinal tract (1). Although the precise etiology of CD remains unclear, several factors are believed to play a role in its development and progression. Given the results from genome-wide association studies (GWAS), it is undisputed that genetic susceptibility plays an important role in disease development (2, 3). Among immune-related conditions, CD is special in that the genetic contribution to disease is high with concordance rates up to 50% among monozygotic twins (4, 5). However, in countries that have adopted a “modernized” lifestyle the incidence rates of IBD have steeply increased in the last few decades. These epidemiological observations clarify that the host genotype alone accounts for a significant but limited proportion of CD risk and rather underline the multifactorial nature of the disease (6–10). The role of the intestinal microbiota in disease pathogenesis has become more and more appreciated. Evidence from genetic, immunological, and microbial studies implicates that chronic intestinal inflammation results from a dysregulated immune response toward components of the commensal intestinal microbiota in genetically susceptible individuals (11). This perception is supported by the identification of several risk loci associated with CD, including genes involved in intracellular processing and killing of bacteria (e.g., NOD2, ATG16L1, IRGM) (12, 13).

Starting with the intestinal lumen, the first part of this review will focus on the intestinal microbiota composition and its changes during inflammation. In the following sections, we will then take a closer look at deeper layers of the intestinal tissue to dissect the interplay of the intestinal microbiota with cells of the host immune system.

## Human Intestinal Microbiota

### Intestinal microbiota – composition

The assembly of the human gut microbiota starts during birth with *Bifidobacterium* and *Lactobacillus* derived from the vaginal canal and breast milk displaying the microorganisms that initially colonize the human intestinal tract (14). During adolescence, phylogenetic diversity of the microbial community increases leading to a complex, diverse, and dynamic microbiota. The adult human gastrointestinal tract contains an abundant microflora, including bacteria, archaea, eukarya, and viruses (15). Recent development of culture-independent molecular profiling methods has greatly advanced our understanding of the microbiome (16). Nowadays, the composition of microbial communities is typically evaluated by targeting the bacterial 16S ribosomal RNA gene as a phylogenetic marker (15). These culture-independent molecular techniques indicate that the human microbiota contains about 200 strains of bacteria comprising over 100 different bacterial species, dominated by just a few phyla (16). Even though these techniques revealed a high level of variability between individuals at the bacterial species level, *Firmicutes* and *Bacteroidetes* represent the predominant phyla across all vertebrates, representing over 90% of all intestinal bacteria. It is assumed that the individual composition of the microbiota is relatively stable over time, a term called “resilience.” Factors that promote microbial diversity in healthy adults include diet, environment, gender, and genetics among others (17, 18). Studies focusing on gut microbiota profiles of adult humans with varying degrees of genetic relatedness effectively demonstrated the impact of genetic and environmental factors on gut microbiota development. The intestinal microbiota composition of monozygotic twins indicated a high degree of similarity, but was yet distinct. Individuals who were living in the same environment and shared similar eating habits showed the least similarity, while siblings displayed an increased similarity in their species profile (1, 19). Recent studies in nucleotide-binding oligomerization domain 2 (*Nod2*)-deficient mice and humans carrying *NOD2* variants point to an essential role of *Nod2* for the development and composition of the host microbiota. *Nod2*-deficient mice displayed an increased load of commensal microbiota and an altered microbiota composition. Additional studies in weaning mice illustrated that NOD2 may affect the microbial community early in life. Furthermore, the substantial changes in the microbiota composition caused by *Nod2* deficiency was paralleled by an increased colitis severity following chemically induced injury. Subsequent co-housing and cross-feeding experiments revealed the transmissibility of the observed genotype-dependent disease risk (20). Taken together, these studies clearly indicated that the host genotype has a lasting effect on the intestinal microbiota composition and *vice versa* the intestinal microbiota is capable of determining the host phenotype.

### Intestinal microbiota – shaping the gastrointestinal immune system

In health, the relationship between the host and the intestinal microbiota provides mutual benefits. On the one hand, the microbiota benefits from the nutrient-rich environment of the gut paving the way for the establishment of a relatively stable ecosystem. In turn, the intestinal microbiota enriches the host with vital functions that the host itself cannot perform. The intestinal microbiota is substantial for mucosal barrier function, affects the development of the mucosal immune system, and is essential for a number of physiological metabolic processes as described further on (21). The profound effects of the commensal microbiota on intestinal and immune cell development have best been highlighted by the engraving phenotype of germ-free (GF) mice. One of the first deficiencies observed in GF mice was a profound reduction of secretory immunoglobulin A (IgA) levels in the intestine. Subsequent mono-association of these mice with various bacteria was shown to lead to an increased IgA expression (22). In addition to numerous defects in antibody production, GF mice display various morphological tissue defects in their intestines [Table 1; (23)]. These developmental impairments are attenuated following the introduction of gut bacteria, once more illustrating the indispensable connection between the ultrastructural development of the intestine and the commensal microbiota. Next to defects in intestinal organ development, investigations on GF mice also revealed cellular defects in intestinal epithelial and lamina propria lymphocytes as well as in mesenteric lymph nodes (Table 1). Normal functioning of intestinal epithelial cells, including the expression of microbial recognition receptors, defensins, and antimicrobial peptides (AMPs), was shown to be impaired in GF animals compared to their conventionally raised counterparts (23).

**Table 1:** **Defects in the intestinal mucosal immune system in GF mice**.

Defects in intestinal organ development in germ-free (GF) mice		

	Site	Phenotype in GF mice
Small intestine	Peyer’s patches	Fewer, less cellular
	Lamina propria	Thinner, less cellular
	Germinal centers	Fewer plasma cells
	Isolated lymphoid follicles	Smaller, less cellular
MLN	Germinal centers	Smaller, less cellular
		Fewer plasma cells

**Cellular defects in germ-free (GF) mice**		

	**Cell type**	**Phenotype in GF mice**

IEL	CD8^+^ T cells	Fewer, reduced cytotoxicity
LPL	CD4^+^ T cells	Proportional decrease in number
	CD4^+^ T cells	Decreased Th17 cells (small intestine)
	CD4^+^ T cells	Increased Th17 cells (colon)
MLN	CD4^+^CD25^+^ T cells	Reduced expression of FoxP3
	CD4^+^CD25^+^ T cells	Reduced suppressive capacity

*IEL, intestinal epithelial lymphocytes; LPL, lamina propria lymphocytes; MLN, mesenteric lymph nodes; GF, germ free [adapted from Ref. (23)]*.

Detailed investigations on certain members of the intestinal microbiota served to unravel mechanisms by which commensal bacteria induce immune tolerance. For example, investigations on segmented filamentous bacteria (SFB) revealed that these Gram-positive bacteria are sufficient to promote T helper 17 cells (Th17) development in the small intestinal lamina propria (24). In addition, colonization of GF mice with SFB resulted in an increased production of serum amyloid A in the terminal ileum, which in turn enhanced IL-6 and IL-23 production by lamina propria dendritic cells (DCs), thereby stimulating a Th17 inducing environment (24). Th17 effector cytokines enhance epithelial cell tight junctions, induce mucin production, and have been associated with induction of AMPs. Colonization of GF mice with SFB resulted in the induction of multiple AMP genes, for example, *RegIII*γ (24). Even though Th17 cells are crucial for protecting the host against pathogenic infection, it should be noted that these cells also display an inflammatory potential as observed in different murine models of autoimmune diseases (25, 26). Recently, certain strains within *Clostridia* clusters XIVa, IV, and XVIII were shown to induce regulatory T cell (Treg) responses in the colon (27). Another prominent human commensal, *Bacteroides fragilis*, was found to direct the development of FoxP3^+^ Tregs via the immunomodulatory molecule polysaccharide A. Monocolonization of GF mice with *B. fragilis* was accompanied by an increased suppressive Treg capacity and the induction of an anti-inflammatory cytokine profile emerging from FoxP3^+^ T cells in the gut (28).

### Intestinal microbiota – metabolic functions

Next to their ability to promote immune system development and maturation as outlined above, the intestinal microbiota enriches the host with metabolic functions by synthesizing vitamins and degrading complex indigestible dietary carbohydrates and proteins. Fermentation of dietary fiber leads to the production of short chain fatty acids (SCFAs), primarily acetate, propionate, and butyrate (21, 29). The functions of SCFAs in promoting colonic health range from displaying a unique energy source for colonocytes to mediating anti-inflammatory and anti-tumorigenic effects (30). Therefore, it is easily comprehensible that conditions coming along with reduced SCFA-levels, including diversion colitis, fiber-free diet, or GF conditions, present with metabolic starvation and consecutive colonic atrophy (31). Among SCFAs, butyrate has received most attention for its effects on colonic health. Butyrate displays an anti-inflammatory effect by decreasing the expression of pro-inflammatory cytokines via inhibition of NF-κB activation and was shown to exert the ability to influence gene expression in the colon through histone deacetylase inhibition (32, 33). Moreover, butyrate elicits biological effects by binding to G protein-coupled receptors, namely GPR109A and GPR43. Butyrate serves as an endogenous agonist for GPR109A, expressed in the colonic epithelium, adipose tissue, and on immune cells, while GRP43 is activated by all three SCFAs (34). The activation of GPR109A results in a decrease of intracellular cAMP-levels accompanied by controlling electrolyte and water absorption, thereby potentially affecting the incidence of diarrhea (35). Activation of GPR109A by butyrate was found to impose anti-inflammatory properties in colonic macrophages and DCs and enabled them to induce the differentiation of Tregs and IL-10 producing T cells. Furthermore, GPR109A signaling was shown to be decisive for butyrate-mediated induction of IL-18 in the colonic epithelium (36). Comparative analysis in GF mice and their conventionally colonized counterparts revealed markedly reduced SCFA concentrations in the intestines of GF mice. Application of SCFAs via drinking water restored colonic Treg homeostasis and function in GF mice, suggesting that their lack of SCFAs may account at least partially for their immune defects, especially their reduced colonic Treg numbers (37). In a murine model of colitis, dextran sulfate sodium (DSS)-induced inflammation in GF mice was ameliorated through additional application of acetate. Conventionally colonized mice lacking Gpr43 showed a markedly increased inflammatory response following DSS application as compared to wild-type (wt) mice. Subsequent acetate application via drinking water resulted in a decrease of colonic inflammation but only in wt mice. Additional acetate application in *Gpr43*^−/−^ mice did not lead to alterations in the severity of inflammation indicating that the protective effect of acetate seen in wt mice occurred through binding to GPR43 (38). Most recently, Mackay and colleagues provided evidence that diet deficient or low in fiber content exacerbates colitis induced by DSS, whereas a high-fiber diet was shown to exert a protective effect. Moreover, it was demonstrated that fiber mediates its protective properties via activating the NLRP3 inflammasome (39).

Given the aforementioned tremendous metabolic functions of the intestinal microbiota, the question raises whether inflammation in peripheral tissues is also influenced by intestinal microbiota-derived metabolites. Recently, Marsland and colleagues found evidence that dietary fermentable fiber and SCFAs are capable of shaping the immunological environment not only in the murine intestine but also in the lung (40). Oral administration of a high-fiber diet was accompanied by increased circulating SCFA levels and concomitant protection against allergic inflammation in the lung following exposure to house mite extract (HM) through intranasal administration. By contrast, feeding mice with a low-fiber diet resulted in decreased SCFA levels and an increase of allergic airway disease in response to HM application. Accordingly, histological sections revealed enhanced eosinophilic and lymphocytic infiltrates in the airways of mice fed a low-fiber diet as well as enhanced concentrations of IL-4, IL-5, IL-13, and IL-17A in lung tissue homogenates. The detected differing outcome in response to HM exposure underlines the impact of SCFAs on shaping the immunological environment in the lung, thereby impinging on the severity of allergic inflammation.

### Intestinal microbiota in disease – changes in the gut microbial ecosystem

Changes in the microbiota composition display a hallmark of CD, commonly described as dysbiosis. Individuals with IBD have been characterized by marked qualitative and quantitative changes in their microbiota composition (41). Several culture-dependent and -independent analyses focusing on the microbiota profile of CD patients revealed less complex microbiota profiles and higher numbers of mucosa-associated bacteria compared to healthy individuals (41–45). Metagenomic-based studies have reported a reduction in members of the phyla Bacteroidetes and Firmicutes in patients suffering from CD or UC (41, 46, 47). Among members of the Firmicutes phyla, significant reductions in the butyrate-producing bacterium *Faecalibacterium prausnitzii* (*F. prausnitzii*) have been observed repeatedly in CD patients (7, 48). This bacterium displays anti-inflammatory properties, which have been intensively studied both *in vitro* and *in vivo*. Stimulation of peripheral blood mononuclear cells with *F. prausnitzii* induced very low levels of the pro-inflammatory cytokines IFNγ and IL-12 paralleled by high levels of IL-10 (48). These *in vitro* effects were confirmed *in vivo* by using a 2,4,6-trinitrobenzenesulphonic acid (TNBS)-induced model of colitis. Oral administration of *F. prausnitzii* markedly reduced the severity of TNBS colitis and tended to rectify the dysbiosis associated with colitis in this model. Follow-up studies after ileal resection provided evidence that a low proportion of *F. prausnitzii* on resected ileal mucosa of CD patients was associated with an increased risk of endoscopic recurrence after 6 months.

The changes in abundance and biodiversity of intestinal microbiota during inflammation are further characterized by an increase of members associated with the Proteobacteria and Actinobacteria phyla. CD patients were shown to harbor increased loads of *Enterobacteriaceae*, in particular *Escherichia coli* belonging to the taxonomic lineages B2 and D (49). Adherent-invasive *E. coli* (AIEC) pathovar has been commonly identified in the intestinal mucosa of patients with CD, particularly associated with ileal mucosal lesions (50, 51). Isolates of these certain strains of *E. coli* were shown to not only adhere to epithelial cells but also to invade and replicate intracellularly. Moreover, AIEC is able to survive and replicate within macrophages without triggering host cell death accompanied by the release of large amounts of tumor necrosis factor (TNF)-α (52). Compared to healthy controls, mucosal biopsy specimens from CD patients displayed a 10-fold higher presence of bacteria that penetrate the mucus layer. These results from fluorescent *in situ* hybridization analyses indicate that in CD the microbiota might have closer contact with the mucosa, a hypothesis that will be discussed in a later section. However, this finding could be explained by the increased numbers of mucolytic bacteria, such as *Ruminococcus gnavus* and *Ruminococcus torques*, observed in macroscopically and histologically unaffected intestinal epithelium of CD patients (53). In 2011, the so far largest cohort study provided a detailed description of the microbiome in CD patients (54). A lower occurrence of *F. prausnitzii*, *Bifidobacterium adolescentis*, *Dialister invisus*, and an uncharacterized species belonging to *Clostridium* cluster XIVa as well as a higher number of *R. gnavus* characterized the dysbiosis signature of CD patients (54). From a metabolic point of view, this comes along with an increased mucolytic and reduced butyrate-producing capacity. Moreover, this study not only confirmed previous findings but also propounded that these changes were markedly characteristic for the disease as this profile was not found in unaffected relatives of analyzed patients.

## Intestinal Microbiota in the Pathogenesis of Crohn’s Disease

### Experimental and clinical evidence

Over the last decades, a consistent body of evidence has accumulated supporting the role of the intestinal microbiota in precipitating IBD. Studies in IL-2- and IL-10-deficient mice were one of the first highlighting the role of the intestinal microbiota in the induction and perpetuation of chronic inflammation. Spontaneous colitis observed in IL-2 deficient mice when raised under specific-pathogen-free (SPF) conditions was unverifiable in animals with the same genetic background bred under GF conditions. Similarly, IL-10-deficient animals developed an attenuated inflammation with regard to disease severity and expansion when kept in a facility with a defined microbial environment (55, 56). Subsequently, *Tbet*-deficient mice were shown to develop chronic colitis with a histopathological similarity to human UC. The study revealed also a correlation of the presence of *Proteus mirabilis* and *Klebsiella pneumoniae* and colitis in these animals. Furthermore, healthy wt mice developed chronic colitis upon co-housing with T-bet^−/−^/Rag2^−/−^ mice (TRUC) or following gavage of feces from TRUC mice (57, 58). These data indicate that loss of T-bet influences bacterial populations to become colitogenic and that colitis is transferable to genetically intact hosts. More recently, studies in TNF^delta ARE^ mice provided clear experimental evidence for the causal role of bacterial dysbiosis in the development of chronic small intestinal inflammation. Deletion in the TNF adenosine–uracil (AU)-rich elements (ARE) leads to TNF-driven spontaneous small intestinal inflammation, characterized by both transmural manifestation and a predominant ileal involvement, thereby mimicking key features of human CD pathology (59). Haller and colleagues assessed the impact of intestinal bacteria in this model of ileitis by using different hygienic conditions (GF, SPF, and conventional housing) as well as antibiotics. CD-like ileitis development was completely absent in GF mice. Antibiotic treatment resulted in an amelioration of disease severity and a recurrence of inflammation was observed following the relapse of microbiota composition. Moreover, transfer of dysbiotic cecal microbial communities from SPF–TNF^delta ARE^ mice into GF recipients resulted in the development of ileitis, mimicking inflammation severity of corresponding donors (60). These aforementioned studies just provide examples for the experimental evidence that has accumulated over the last years.

Event though CD can manifest anywhere along the human alimentary tract, it is mainly observed in areas containing the highest concentrations of bacteria (i.e., terminal ileum and colon). One of the first clinical references for the involvement of the intestinal microbiota in disease pathogenesis came from experiments showing that diversion of the fecal stream from an inflamed segment of the small intestine improved symptoms of CD patients. Furthermore, restoration of fecal stream and postoperative exposure of the neoterminal ileum to luminal contents induced inflammation, indicating that the microbiota acts as a trigger in postoperative recurrence of CD (61, 62). Numerous arguments in favor of the involvement of the human microbiota in disease pathogenesis have emerged over the last years (Table 2).

**Table 2:** **Clinical evidence – involvement of the human microbiota in Crohn’s disease pathogenesis**.

Arguments on behalf of the involvement of intestinal microbiota in CD	Reference
Feacal stream diversion improves symptoms of CD	(62)
Reinfusion of luminal contents results in recurrent disease	(61)
Antibiotic therapy is associated with clinical improvement	(63–65)
Mucosal barrier defects and increased translocation	(66, 67)
Higher loads of mucus-associated bacteria	(42)
Higher concentrations of mucolytic bacteria	(53)
Decrease in *Faecalibacterium prausnitzii*	(41, 46, 48)
Decreased concentrations of AMP	(68)
CD susceptibility genes: involvement in killing of intracellular bacteria and secretion of AMP	(69–75)
Siblings of CD patients exhibit mucosal dysbiosis	(76)

*CD, Crohn’s disease; AMP, antimicrobial peptides*.

Although there has accumulated a large body of experimental and clinical evidence supporting the role of dysbiosis in the pathogenesis of CD, it is still up for discussion whether these alterations in the gut microbiota composition represent a cause or a consequence of chronic intestinal inflammation. Taking into account that the development of inflammation was shown to be dependent on the presence of a gut microbiota in different murine models and given the insufficient data to recommend probiotics for use in CD implies a pathogenetic role (55, 56, 77, 78). Investigations on fecal microbiota composition in first-degree relatives of patients with IBD have coined the term “predysbiosis.” As already outlined above, several studies indicated a significant impact of the host genotype on the composition of the intestinal microbiota. Relatives of CD patients are at much higher risk of developing CD as compared with the general population. The risk of falling ill is highest in first-degree relatives, especially siblings, and also extends to more distant relatives (54, 79). The relative risk of developing CD was shown to be over 30-fold higher in siblings of patients with CD compared to that of the general population (76). Moreover, an over 50-fold increase in the incidence of IBD was reported within multiply affected families (80, 81). Unaffected relatives of CD patients were found to have a different composition of their intestinal microbiota compared to healthy controls without familial predisposition (54). This subclinical dysbiosis observed in asymptomatic relatives was different from the dysbiosis detected in CD patients and was characterized by lower numbers of both *Collinsella aerofaciens* and an unspecified member of the *E. coli – Shigella* group as well as higher numbers of the mucolytic bacterium *R. torques* compared with healthy subjects. *R. torques*, a non-butyrate-producing member of the *Clostridium* cluster XIVa is capable of degrading gastrointestinal mucin. In contrast to the dysbiosis detected in CD patients, the subclinical dysbiosis in their relatives was not characterized by a diminished butyrate-producing capacity but enhanced mucin degradation might be assumed in these individuals. Given the differences between the observed dysbiosis in CD patients and the subclinical dysbiosis in unaffected relatives, the results from this cohort study suggest that a “predysbiosis” may precede the clinical manifestation of CD. Moreover, the increased numbers in mucolytic bacteria let one suppose that an enhanced mucin degradation capacity of the intestinal microbiota represents an interim step, leading from normobiosis to the investigated dysbiosis among the patient cohort (54). In a recently published study, Hedin and colleagues focused on the mucosal microbiota in healthy siblings of CD patients and described a mucosal dysbiosis characterized by a reduced diversity of core microbiota and a lower abundance of *F. prausnitzii* in these at risk individuals. The lower abundance of *F. prausnitzii* is also one of the convincingly observed species-specific findings in CD patients’ dysbiosis, therefore also suggesting the causative role rather than being a consequence of chronic inflammation (76). Further arguments in favor for an etiological contribution of dysbiosis in CD are provided by the transmissibility of inflammation to genetically susceptible hosts as demonstrated in numerous *in vivo* models. However, similar patterns of microbial changes in diverse hosts as well as the fact that inflammation *per se* is capable of inducing dysbiosis pleads for the theory of dysbiosis as a consequence of the inflammatory milieu. The fact remains that the altered microbial composition entails metabolic consequences and subsequent changes in the intestinal milieu deriving from the variety of essential functions provided by the intestinal microbiota and the crosstalk between the microbiota and the host immune system.

## Host–Microbiota Crosstalk – Innate Mechanisms for Maintaining Intestinal Homeostasis

The innate immune system consists of the intestinal epithelium and cells of the innate immune system, such as neutrophils, DCs, monocytes/macrophages, and innate lymphoid cells (ILCs). The luminal surface of the gastrointestinal tract with approximately 300–400 m^2^ represents the largest interface between the host and the environment. The diverse and abundant indigenous microflora exists in close proximity to the immune system of the intestinal mucosa and immune cells in the underlying tissue. Moreover, the gastrointestinal mucosa is continuously exposed to antigens derived from the food and has to deal with pathogenic microorganisms that can cause tissue damage. The intestinal immune system therefore possesses multiple layers of protection to respond against invading bacteria, thereby limiting their exposure to the systemic immune system while maintaining tolerance toward luminal bacterial antigens and food antigens. Given that a dysregulated immune response toward components of the intestinal microbiota is thought to be a fundamental pillar of chronic intestinal inflammation development, the complex interaction of the host with the plentiful intestinal microbiota has to be precisely regulated.

### Barriers of protection

#### The Intestinal Mucus Layer – A Protective Blanket

The intestinal epithelium lies at the interface between the intestinal lumen and the gastrointestinal-associated lymphoid tissue, thereby building the first barrier against excessive microbial translocation to the lamina propria (82). The intestinal epithelium itself is covered with a mucus layer constituting the first physical barrier to luminal antigens. Mucus is secreted by goblet cells and typically contains several major components, which in the intestine comprise of MUC2 and MUC5AC. These densely glycosylated proteins are resistant to digestive enzymes and give the mucus its gel-like properties (7). The type of mucus organization in the small and large intestine are clearly different. The small intestinal mucus fills the luminal space between the villi and is not attached to the epithelial surface. Antimicrobial products such as AMPs derived from epithelial cells and Paneth cells as well as IgA interfuse with the mucus secreted from the crypts, thereby generating an antibacterial gradient that acts to keep luminal bacteria away from epithelial surfaces. Of note, the small intestinal mucus layer is penetrable to bacteria but still provides a diffusion barrier (83). The mammalian large intestinal mucus is organized in a two-layer system. The loose outer layer of the mucus is composed of mucin, diluted antimicrobials, and is the normal habitat for commensal bacteria, whereas the inner layer is firmly attached to the epithelial cells, rich in antimicrobials, and displays a low bacterial density (84). The physiological relevance and protective function of the mucus layer has best been highlighted in studies using MUC2^−/−^ mice. Muc2-deficiency was shown to lead to colonic inflammation accompanied by the presence of bacteria in direct contact with the intestinal epithelium (85). Moreover, these animals displayed a much higher susceptibility to infection by pathogens (86). From studies in GF mice, it emerged that intestinal microbiota have a role in shaping the colonic mucus barrier (87). Mice housed under GF conditions have been characterized by an extremely thin adherent colonic mucus layer. Exposure to bacterial products, such as lipopolysaccharide or peptidoglycan, leads to the re-storage of the mucus thickness to levels observed in conventionally housed mice. A recently published work reinforced the assumption that intestinal bacteria affect the host mucus barrier properties. In this study, two colonies of C57BL/6 mice were housed and bred in different rooms but both under SPF conditions. Analysis of the microbiota composition by 16S rRNA gene sequencing revealed significant differences at multiple taxonomic levels between the two separately housed colonies. Interestingly, the two colonies not only differed in their microbiota composition but also have a different mucus phenotype that was shown to be specific for each colony. Whereas the thickness of the mucus layer was similar in both groups, a major difference regarding permeability was detected. One colony displayed a mucus layer impenetrable to bacteria, the other colony had an inner mucus layer that was penetrable to both bacteria and beads. The causal role of the intestinal microbiota in the detected mucus phenotypes was underpinned by subsequent experiments showing that the different mucus properties were transmissible by transfer of cecal microbiota to GF mice (88). Although genetic risk loci in *MUC1* and *MUC19* have been identified in IBD, there is only limited and widely varying data regarding changes of the mucus layer during CD (3). One study investigated the thickness and continuity of the mucus barrier in rectal biopsies from CD patients but only provided a small number of cases (89). In this study, no significant differences between CD patient specimens and controls were found. Another study reported a depletion of the mucus layer at inflamed sites of the gut of CD patients compared to healthy controls as well as compared to non-inflamed areas in the same patient (90). Of note, there were no significant differences regarding the thickness of the mucus layer between non-inflamed areas of CD patients and healthy controls. These results are in direct contrast to previously published data measuring the thickness of the colonic mucus in surgically resected specimens from CD patients. Pullan and colleagues reported increased values compared to healthy controls (91). As briefly outlined above, the colonic mucus is organized in two layers. Given the contrary results, one has to consider that in the study showing a thicker mucus layer in CD patients a PAS/AB staining technique was used. During PAS/AB staining variable amounts of the loose outer mucus layer might remain thus contributing to the larger thickness observed in CD specimens. The different results might also reflect different stages of disease and moreover, inflammation in CD is characterized by a discontinuous appearance therefore a general mucus barrier dysfunction is unlikely to occur.

#### Antimicrobial Peptides and Antibodies

A substantial mechanism for controlling the contact between intestinal epithelial cells and luminal antigens is the secretion of AMPs. One of the intensive studied proteins among AMPs is the antibacterial lectin *RegIII*γ produced by multiple epithelial lineages, including enterocytes and Paneth cells and capable of mediating direct killing of Gram-positive bacteria (92). Mouse *RegIII*γ and its human counterpart HIP/PAP are primarily expressed in the small intestine. Inflammatory conditions were shown to increase the expression of *RegIII*γ in the mouse intestine, likewise HIP/PAP expression is increased in the mucosa of IBD patients (93). Reconstitution of GF mice with an intestinal microbiota from conventionally raised mice resulted in a sharp increase in the abundance of *RegIII*γ transcripts in Paneth cells (92). This study also explored the mRNA expression of *RegIII*γ during weaning reflecting an early stage of intestinal development. *RegIII*γ mRNA levels showed a sharp upward movement that was missing in GF controls. To further unravel the host–microbial interactions underlying the regulation of *RegIII*γ, Cash and colleagues investigated the dynamic of this antibactericidal protein in mice lacking IgA. *Bacteroides thetaiotaomicron* and *Listeria innocua* – under state conditions compartmentalized in the intestinal lumen – showed an increased mucosal adherence in the absence of IgA and were found to substantially trigger a vast *RegIII*γ mRNA increase in GF mice lacking IgA. These results indicate that an increased contact between microbiota and the intestinal epithelium drives *RegIII*γ expression. The role of *RegIII*γ as a key element of the intestinal mucosal defense has further been validated in murine *RegIII*γ-deficiency studies that revealed an increased bacterial colonization of the intestinal epithelial surface (94).

Another major group of AMPs is represented by α-defensins, largely produced by small intestinal Paneth cells and β-defensins produced by most epithelial cells (95). Human defensin (HD)-5 and HD-6, both belonging to the group of α-defensins as well as the human β-defensin (HBD)-1, are expressed constitutively (16). HBD-2 and HBD-3 belong to the group of inducible defensins and are expressed in the large intestine. The induction was shown to be mediated by pro-inflammatory cytokines, such as IL-1β, involving NF-κB pathways (96, 97). Induction was also observed following the recognition of intestinal bacteria by pattern recognition receptors (PRRs) (98). Decreased defensin levels can result in a weakening of the intestinal epithelial barrier and might be involved in the pathophysiology of chronic inflammation. Ileal CD is associated with reduced expression of Paneth cell-derived α-defensins and colonic CD was reported to be associated with reduced expression of β-defensins by enterocytes (68). Next to their antimicrobial property, α-defensins possess an additional homeostatic role in regulating and shaping the composition of the small intestinal microbiota. Transgenic expression of DEFA5 (α-defensin) comes along with a significant decrease of members of the phylum Firmicutes, paralleled by an increase in the percentage of Bacteroidetes. Moreover, transgenic expression of DEFA5 also causes a loss of SFB and concomitantly Th17 in the lamina propria (99).

A third immune mechanism serving to control the microbiota and reduce the contact between intestinal bacteria and epithelial cells is the secretion of IgA by lamina propria plasma cells (100). DCs within the Peyer’s patches and subepithelial dome region continuously sample luminal bacteria. Bacteria- and antigen-loaded DCs interact with and prime T- and B-cells in the Peyer’s patches or find their way to the gut-draining mesenteric lymph nodes via the afferent lymphatics. The interaction of antigen-bearing DCs with naïve B cells induces the activation and differentiation of naïve B cells to IgA producing plasma cells. These plasma cells leave the Peyer’s patches or mesenteric lymph nodes, respectively, enter the systemic circulation via the efferent lymphatics and return to the intestinal lamina propria where they secrete IgA into the intestinal interstitium. The secreted IgA is specific for commensal microbiota, subsequently taken up by epithelial cells and delivered to their apical surface. When released into the intestinal lumen, IgA binds to commensal microbiota, thereby limiting their capability to penetrate the epithelial barrier (7, 101). Remarkably, live bacteria transported by DCs do not gain access to the systemic circulation at any time thus are not able to induce systemic immune responses. Recent studies provide evidence that goblet cells, supposedly purely secretory cells, also act as luminal sensors for the innate immune system. McDole and colleagues were able to show that in the absence of inflammation, small intestinal goblet cells act as passages delivering low molecular weight soluble antigens from luminal to CD103^+^ DCs in the underlying lamina propria (102). The interconnection between goblet cells and DCs with tolerogenic potential illustrate that goblet cells contribute to the communication between luminal antigens and the innate immune system.

Taken together, these aforementioned innate barriers of protection are crucial for intestinal homeostasis. Moreover, a finely balanced interplay between the intestinal microbiota and cells of the host immune system is indispensable for ensuring the functionality of these barrier mechanisms.

#### Epithelial Barrier and the Crosstalk Underneath

In contact with the inner mucus layer, there is the intestinal epithelium consisting of four main types of epithelial cells, namely enterocytes, enteroendocrine cells, goblet cells, and Paneth cells. The intestinal epithelium contributes to absorption, digestion as well as secretion, and functions as a mucosal barrier (84). Mucosal barrier integrity is maintained by tight junctions, desmosomes, and adherence junctions. Impairment of the epithelial barrier is accompanied by increased intestinal permeability and bacterial translocation leading to persistent immune activation, a condition that has been observed in both CD and UC patients (103). In health, only small amounts of luminal antigens are allowed to pass across the epithelium. Murine models displaying an impaired barrier function were shown to develop intestinal inflammation. Junctional adhesion molecule-A (JAM-A) is a key structure of tight junctions and mandatory for controlling cell migration into the underlying tissues. Studying of CD tissue specimens for JAM-A expression revealed a loss of epithelial JAM-A expression (104). In line with these findings are the decreased levels of further tight junction proteins, such as claudins, observed in CD (66).

Lying between the luminal microbiota on the one side and immune cells in the lamina propria on the other, the intestinal epithelium functions to communicate with both. Sensing of microbial antigens by epithelial cells as well as by innate immune cells, such as macrophages and DCs, is crucial for maintaining intestinal homeostasis and is mediated by PRRs (95). These receptors include the family of toll-like receptors (TLRs) as well as intracytoplasmatic receptors, such as NOD-like receptors (NLRs). Polymorphisms in TLRs and NLRs have been implicated in increased susceptibility to IBD (3, 69). Whereas TLRs are capable of detecting a variety of bacterial components, such as lipopolysaccharide, lipoproteins, and CpG DNA, NLRs recognize peptidoglycan molecules on the bacterial cell wall (105). Activation of PRRs results in down-stream signaling cascades that are widely mediated by *Myd88*. *MyD88* represents a cytosolic adaptor protein, equipped with a toll/IL-1 receptor (TIR) domain, thereby able to directly bind TLRs (106). Once engaging TLRs, *MyD88* mediates MAP kinase activation, recruitment of a variety of signaling molecules and drives nuclear translocation of NF-κB and subsequent production of pro-inflammatory cytokines. Given the abundance of commensal antigens in the intestinal lumen, it is crucial that activation of PRRs not exclusively drives inflammatory responses. Excessive stimulation can lead to detrimental inflammation. Otherwise, repetitive stimulation of TLRs due to commensal bacterial exposure was shown to result in a down-regulation of the NF-κB pathway and stimulation of AMP production (1). Moreover, triggering of PRRs also promotes antigen presenting cell maturation and is involved in proliferation of Tregs (107). Already about one decade ago, Rakoff-Nahoum and colleagues provided experimental evidence that the recognition of the commensal microbiota by TLRs is crucial for maintenance of intestinal epithelial homeostasis and required for regeneration after mucosal injury (108). TLR expression and activation has to be tightly regulated and this regulation is exerted by a number of mechanisms, such as adjustment of their expression, their localization, and their positioning within the tissue. Most surface TLRs are principally found on the basolateral side of IEC and are only up-regulated during inflammation (109).

Punctilious sampling and processing of luminal antigens is essential for avoiding the development of intestinal inflammation and establishing and sustaining a healthy relationship between the commensal microbiota and the host. DCs are an indispensable element in this crosstalk as they are continuously exposed to antigens derived from commensal microbiota, dietary products, and intestinal pathogens. The ductility of DCs is warranted by their ability to adapt to influences of the microenvironment. In this context, different DC populations with different functionality have been described. Gut-resident DCs display tolerogenic functions as they encounter and sense commensal bacteria and act, for example, by inducing a highly tolerogenic response through differentiation and expansion of Tregs (110, 111). The triangular crosstalk between epithelial cells, DCs, and the intestinal microbiota is orchestrated by PPR signaling. Using an *in vitro* model, intestinal epithelial cells were shown to release cytoprotective factors, such as thymic stromal lymphopoietin and transforming-growth factor-β upon activation of TLR-signaling, leading to the presence of a predominantly tolerogenic DC phenotype. These tolerogenic DCs were found to secrete IL-10, leading to an immune response dominated by Tregs (112).

Even though the intestinal epithelium with its numerous lines of protection provides a barrier separating luminal antigen from the underlying tissue, it is inevitable that antigens gain access to the lamina propria. As mentioned above, this might not only occur through injury or infection but also happens when commensals undergo epithelial cell transcytosis or translocation via M cells or DCs (113). In response to transforming-growth factor-β and IL-8, chemokines abundant in the extracellular matrix of the lamina propria, circulating blood monocytes gather to the non-inflamed lamina propria where they become resident intestinal macrophages. Located strategically in the subepithelial lamina propria, intestinal macrophages are part of the armament of innate defense mechanisms. The interaction between macrophages and microorganisms is mediated by PRRs, including TLRs and NLRs. In contrast to peripheral macrophages, intestinal macrophages were shown to perform their defense activities without releasing inflammatory cytokines while retaining their phagocytic and bacteriocidal activity. Consequently, intestinal macrophages do not provoke a pro-inflammatory cytokine milieu in the non-inflamed mucosa despite the close proximity to and interaction with immunostimulatory microorganisms. This so-called “inflammatory-anergy” was shown to be at least in part attributable to matrix-bound transforming-growth factor-β and Tregs causing a down-regulation of PRRs and related adapter proteins, resulting in NF-κB inactivation (113, 114). In the case of inflammation or intestinal infection, blood monocytes accumulate in the lamina propria and actively combat invading microorganisms through uptake and degradation, including the release of inflammatory mediators.

Over the last decade, an emerging family of innate immune cells, termed ILCs has been characterized. Since ILCs reside primarily at mucosal sites and are enriched at mammalian barrier surfaces, including the intestine, these cells are in close proximity to environmental antigens and commensal microbiota in particular. Commensal microbiota were found to provide signals for the development, differentiation, and function of ILCs and this crosstalk, the interplay between the microbiota and ILCs is indeed an area of intense research. ILCs have been shown to contribute to tissue repair and remodeling at barrier surfaces and were found to influence inflammatory conditions, thereby orchestrating host–commensal relationships (115). Being derived from an Id2-dependent lymphoid progenitor cell population, ILCs are of lymphoid origin but do not require antigen receptors generated by somatic recombination (116). Based on transcription factors required for their development, their cytokine production patterns and surface markers, the family of ILCs is categorized in three main branches. In brief, group 1 ILCs (ILC1) are Tbet- dependent and are composed of ILC1s and NK cells. ILC1s respond to IL-12 and IL-18 and produce IFN-γ. Group 2 or GATA3^+^ ILCs secrete IL-5, IL-9, IL-13, and amphiregulin in response to IL-33 and IL-25. Finally, group 3 ILCs (ILC3) express RORγt and include lymphoid tissue-inducer (LTi) cells and natural cytotoxicity receptor (NCR) expressing ILCs. Group 3 ILCs respond to IL-1β and IL-23 and are the main producers of IL-17 and IL-22 (117). Given the spatial proximity of ILCs and commensal microbiota in the intestine, multiple groups have investigated the possible role for commensal microbiota in the development of ILCs. Studies using GF mice indicated that NK cells and GATA3^+^ ILCs develop properly without receiving signals from commensal microbiota (118, 119). Regarding RORγt^+^ ILCs, there have been controversial reports on the requirement of commensal microbiota for their development. It seems that the development of at least subsets of RORγt^+^ ILCs occurs regardless of the presence of a commensal flora as evident by the presence of LTi cells as well as the generation of secondary lymphoid structures in the sterile environment before birth (115, 120, 121). However, the ripening of intestinal cryptopatches into isolated lymphoid follicles was found to be compromised in GF mice, suggesting at least a functional impairment in some populations of LTi-like RORγt^+^ ILCs (122). Several groups reported normal development of RORγt^+^ ILC subsets in both GF and antibiotic treated mice (115, 123, 124). By contrast, other studies proposed that commensal bacteria are capable of enhancing the development of NCR^+^ RORγt^+^ ILCs and IL-22 production. IL-22 produced by this population in turn contributed to epithelial homeostasis by regulating genes involved in tissue repair and antimicrobial defense mechanisms, such as *RegIII*β and *RegIII*γ (125–127). Factors accounting for these differing results may relate to host genetics, a differential presence of other cell types and cytokines and not least to a varying exposure to non-live bacterial- or diet-derived signals. Although it seems that the development of most ILCs is not reliant on bacterial interaction, signals derived from the commensal microbiota directly affect the function of ILCs. The regulation of ILC function by commensal bacteria can happen indirectly or directly through engagement of PRRs on ILCs themselves. For instance, human NK cells were shown to express functional TLR2 and TLR9 (128, 129). Moreover, stimulation of human RORγt^+^ ILCs with TLR2 agonists resulted in IL-2 production which in turn enhanced IL-22 expression (130). In addition, there is accumulating evidence that NCRs of NK cells and NCR^+^ RORγt^+^ ILCs act as direct sensors of components of the commensal bacteria (131–133). Next to direct signaling through TLRs and NCRs, different groups provided evidence that the aryl hydrocarbon receptor (AhR), expressed by RORγt^+^ ILC3s and well known for its critical function in ILC development and IL-22 production has also a role in the commensals regulatory function. More specifically, the AhR can be stimulated by ligands derived from the tryptophan metabolism of commensal microbiota, thereby having an intermediate position between direct and indirect regulation (134). In addition to direct regulation, commensal bacteria also take advantage of an indirect route of influencing the function of ILCs via triggering cytokine signals from epithelial cells or other innate immune cells. As one example, commensal bacteria captured by DCs lead to the induction of IL-12 that acts on ILC1s by stimulating their IFN-γ production that in turn accelerates macrophage phagocytosis (116). Next to the effects of the commensal microbiota on ILCs, there is evidence that ILCs are capable of influence the microbial community. This reciprocal interaction is, for example, simply attributed to signals derived from ILCs that act on the intestinal permeability and thus affect bacterial translocation.

Due to displaying a major source of IL-17 and IL-22, ILC3s are supposed to be critical for the promotion of inflammation and tissue repair in the intestine as well as for dealing with extracellular bacteria (135). Murine models provided evidence that NCR^+^ ILC3s rapidly response to infection with the enteric pathogen *Citrobacter rodentium* by producing IL-22, which is essential for host protection. This ILC3 response is promoted by the occurrence of a positive feedback loop stimulating DCs to the production of both lymphotoxin-β and IL-23 (124, 136). IL-17 and IL-22 derived from ILC3s promotes neutrophil recruitment to the intestine and stimulates *inter alia* the production of RegIIIγ, RegIIIβ, as well as mucus production, thereby also pointing out the important role of ILC3s in tissue repair (135, 137). Moreover, ILC3s were shown to have the potential to regulate not only innate but also adaptive immune responses, a capacity that might be crucial especially with regard to chronic inflammatory disorders like IBD. Mortha and colleagues recently reported that ILC3s are the primary source of granulocyte-macrophage colony-stimulating factor (GM-CSF) in the intestine. GM-CSF production was depend on and regulated by the ability of macrophages to sense commensal microbiota and produce IL-1β. ILC3-derived GM-CSF influenced myeloid cell homeostasis and was essential for the generation of subsequent Treg responses toward food antigens and maintenance of oral tolerance (138). Furthermore, mouse and human ILC3s were also found to express MHCII, and directly interact with CD4^+^ T cells. Most interestingly, murine studies revealed that depletion of ILC3-intrinsic MHCII leads to the development of spontaneous CD4^+^ T cell-driven microbiota-dependent inflammation (139, 140).

Of note, this only provides a brief insight into the complex world of ILCs but states incontrovertibly that the emerging family of ILCs is essential in the context of the crosstalk between the microbiota and the innate immune system, thereby displaying a key determinant in regulating the host–commensal relationship.

## Conclusion

The innate immune system provides multiple layers of protection to regulate and control interactions between the intestinal microbiota and the host. However, the functionality of these protective mechanisms depends on and is positively affected by finely balanced signals derived from the commensal microbiota, thereby ensuring their reliability and performance. In CD, these mechanisms of defense and tolerance are impaired at multiple levels. Intestinal dysbiosis and concomitant changes of the intestinal luminal milieu and environment weaken the intestinal epithelial barrier and entail an increased epithelial permeability. Epithelial barrier dysfunction is followed by an increase of translocation to the lamina propria, where defective handling of antigens might elicit a strong inflammatory response, maintained and enhanced by ineffective phagocytosis, and bacterial clearance as well as impairment of adaptive immune responses. Although it remains unclear, if dysbiosis precedes disease or results from active inflammation, the changes observed during active CD have a lasting and debilitating effect on the numerous host mechanisms that function to maintain homeostasis. Two recent randomized controlled trials provide additional insight by indicating that re-establishing of the intestinal microbiota composition by fecal microbiota transplantation (FMT) ameliorates active UC in a patient subgroup. The first trial conducted by Moayyedi et al. randomly assigned patients with active UC to receive either FMT via enema from healthy anonymous donors or placebo (water via enema) once weekly for a period of 6 weeks (141). The second study also investigated the therapeutic impact of FMT in patients with UC (142). Remarkably, in this study by Rossen et al., autologous fecal microbiota served as control and the administration route of FMT was via nasoduodenal tube. In brief summary and without discussing all study characteristics and statistical outcomes in detail, the decisive insight obtained from these studies is that responsiveness to FMT was accompanied with changes in the microbiota composition and especially with a significant increase of the patients microbial diversity. Moreover, patients with a recent diagnosis of UC seem to be more likely to respond to FMT. Given that seven of the nine responding patients in the study conducted by Moayyedi et al. received fecal material from the same single donor, this leads to the suggestion of a donor-dependent effect and underlines the relevance of the microbiota composition in intestinal homeostasis. Furthermore, it points out that intestinal dysbiosis – irrespective of being cause or consequence – comes along with changes in the intraluminal milieu and together with a genetic predisposition might be capable of triggering a vicious cycle resulting in an abnormal inflammatory response.

This increasing evidence on the interplay of the intestinal microbiome, barrier, and intraepithelial as well as mucosal immune cells displays a complex network that is tightly regulated. Nevertheless, the data discussed indicate several target structures that require in-depth exploration, such as targeting the luminal site via a defined change in the microbiota composition or via selected nutritional restriction that strengthen barrier function and exert anti-inflammatory effects on immune cells. FMT will have to be replaced by a defined administration of a bacterial mix. The definition of this “mix” or additional food compounds that influence the mucosal balance will be the center of research and might ultimately provide novel therapeutic targets not only for IBD.

## Conflict of Interest Statement

The authors declare that the research was conducted in the absence of any commercial or financial relationships that could be construed as a potential conflict of interest.
